# Amniotic Membrane Mesenchymal Cells-Derived Factors Skew T Cell Polarization Toward Treg and Downregulate Th1 and Th17 Cells Subsets

**DOI:** 10.1007/s12015-014-9558-4

**Published:** 2014-10-28

**Authors:** Stefano Pianta, Patrizia Bonassi Signoroni, Ivan Muradore, Melissa Francis Rodrigues, Daniele Rossi, Antonietta Silini, Ornella Parolini

**Affiliations:** 1 Centro di Ricerca E. Menni, Fondazione Poliambulanza-Istituto Ospedaliero, Via Bissolati, 57, I-25124 Brescia, Italy; 2 Doctoral School of Molecular Medicine, University of Milan, Milan, Italy

**Keywords:** Mesenchymal stromal cells, Human amniotic membrane mesenchymal cells, Human placenta, Conditioned medium, Secretome, Immunomodulation, Cytokines, T cells, Th1, Th2, Treg, Th17

## Abstract

We previously demonstrated that cells derived from the mesenchymal layer of the human amniotic membrane (hAMSC) and their conditioned medium (CM-hAMSC) modulate lymphocyte proliferation in a dose-dependent manner. In order to understand the mechanisms involved in immune regulation exerted by hAMSC, we analyzed the effects of CM-hAMSC on T-cell polarization towards Th1, Th2, Th17, and T-regulatory (Treg) subsets. We show that CM-hAMSC equally suppresses the proliferation of both CD4^+^ T-helper (Th) and CD8^+^ cytotoxic T-lymphocytes. Moreover, we prove that the CM-hAMSC inhibitory ability affects both central (CD45RO^+^CD62L^+^) and effector memory (CD45RO^+^CD62L^−^) subsets. We evaluated the phenotype of CD4^+^ cells in the MLR setting and showed that CM-hAMSC significantly reduced the expression of markers associated to the Th1 (T-bet^+^CD119^+^) and Th17 (RORγt^+^CD161^+^) populations, while having no effect on the Th2 population (GATA3^+^CD193^+^/GATA3^+^CD294^+^cells). T-cell subset modulation was substantiated through the analysis of cytokine release for 6 days during co-culture with alloreactive T-cells, whereby we observed a decrease in specific subset-related cytokines, such as a decrease in pro-inflammatory, Th1-related (TNFα, IFNγ, IL-1β), Th2 (IL-5, IL-6), Th9 (IL-9), and Th17 (IL-17A, IL-22). Furthermore, CM-hAMSC significantly induced the Treg compartment, as shown by an induction of proliferating CD4^+^FoxP3^+^ cells, and an increase of CD25^+^FoxP3^+^ and CD39^+^FoxP3^+^ Treg in the CD4^+^ population. Induction of Treg cells was corroborated by the increased secretion of TGF-β. Taken together, these data strengthen the findings regarding the immunomodulatory properties of CM-hAMSC derived from human amniotic membrane MSC, and in particular provide insights into their effect on regulation of T cell polarization.

## Introduction

The human amniotic membrane, as well as the other perinatal tissues, have recently attracted much attention in regenerative medicine applications [1]; indeed they can be easily obtained in a non-invasive manner from tissues normally discarded after birth, and they also offer an abundant source for bank development. The therapeutic potential of the perinatal stem cells has been prevalently associated to their immunomodulatory capacities [2–6] and consequent paracrine effects, as observed in different animal models of disease [7–9].

Amongst perinatal tissues, the human amniotic membrane from term placenta has been recently recognized as a valuable source of mesenchymal stromal cells, referred to as hAMSC [10–12]. Interestingly, studies have shown the ability of hAMSC to interact with and modulate the functions of a wide variety of immune cells. For example, we and others have shown that hAMSC can inhibit T cell proliferation in vitro induced by alloantigens, T-cell receptor cross-linking, or mitogens [13–17]. Furthermore, we and others have previously shown that cells derived from the human amniotic membrane strongly inhibit the generation, maturation, and function of monocyte-derived dendritic cells (DCs) in vitro [18, 19]. The in-vitro anti-inflammatory potential of amniotic cells is in line with the in vivo findings showing reduction of inflammation and fibrosis in animal models of disease following the transplantation of cells derived from the amniotic membrane. For example, therapeutic effects have been observed in bleomycin-challenged mice as shown by a reduction in lung fibrosis following treatment with amniotic cells [5, 20]. Moreover, amniotic cells have been reported to ameliorate prognosis of autoimmune diseases such as rheumatoid arthritis, encephalomyelitis [6], and experimental autoimmune myocarditis [21]. Furthermore, the use of amniotic membrane *patches* were also able to attenuate disease progression. The transplantation of non-cryopreserved amniotic patches [22], or even those after cryopreservation [23], were able to improve liver fibrosis in rats with bile-duct ligation and promote ischemic heart repair in rats with coronary artery ligation [24]. Interestingly, in these studies therapeutic effects were observed despite absence or rare presence of transplanted cells in host tissues. These findings have reinforced their capacity to exert paracrine effects inducing tissue repair by immunomodulation rather than cell differentiation [11]. Confirmation that the molecules released from cells are the key players comes from studies showing that the conditioned medium exerts the same anti-inflammatory effects as cells [25, 26]. Evidence suggests that the conditioned medium obtained from the culture of AM patches or hAMSC inhibits T cell proliferation [27], inhibits the differentiation of monocytes towards DCs, and induces a shift toward M2-like macrophages [28] as observed with MSC from other placental regions [29]. The molecules and mechanisms involved are still unclear, but there are many hypotheses which also take into consideration what is known on mesenchymal stromal cells derived from bone marrow, which have been reported to act through IDO, NO, PGE2, TGF-β, IL-10, HGF and galectins [30, 31]. Moreover, we have provided evidence that this effect seems to be mediated by low molecular weight, non-protein, thermostable compounds present in conditioned medium, and that prostaglandins are one of the key effector molecules in the immunomodulatory activity [27]. Arising from the need to identify key effector molecules is the desire to understand the cells on which they act, and in turn how they are impacted. Specifically, even though the anti-proliferative effects on T cells are now widely accepted, the effects of hAMSC on the different T cell subpopulations remain to be clearly addressed. Recent studies report the capacity of amniotic mesenchymal stromal cells to regulate T cell subsets in animal models. For example, systemic administration of hAMSC has been shown to ameliorate experimental autoimmune myocarditis (EAM) via the suppression of Th1/Th17 immunity [21]. Similar mechanisms have been extensively described for mesenchymal stromal cells obtained from other sources. For example, treatment with bone marrow MSC was shown to attenuate cutaneous delayed-type hypersensitivity in mice and was found to be associated with reduced CD4^+^ and CD8^+^ T cell infiltration at the challenge site [32]. Moreover, the treatment of colitic mice (model of inflammatory bowel disease) with MSC from adipose tissue reduced the Th1 cell responses and induced T regulatory cells [33], while treatment with MSC from bone marrow prevented Th1-mediated autoimmune diabetes mellitus in rats, and was associated with increased CD4^+^ and CD8^+^ FoxP3^+^ T cells [34]. We have very recently demonstrated that treatment of mice with collagen-induced arthritis using cells from the amniotic membrane impaired antigen specific Th1/Th17 cell expansion in the lymph nodes, and generated peripheral antigen-specific T regulatory cells [6]. Taken together, these studies indicate that amnion-derived cells and its conditioned medium do indeed act on T cells. Nevertheless, a basic lack of information regarding the effects that hAMSCs have on individual T-cell effector subsets remains. In this study, we set out to clarify the polarization of T cells by performing detailed in vitro studies on both CD4 and CD8 lineages and we contribute to the understanding of the time-dependent effects on the polarization of CD4^+^ T cells in terms of T cell activation, proliferation, and cytokine production.

## Materials and Methods

### Ethics Statement

Human term placentas were collected after obtaining written informed consent according to the guidelines of the Ethical Committee of the Catholic Hospital (CEIOC, Parere 16/2012) and of the Ethical Committee of the Hospital Fondazione Poliambulanza-Istituto Ospedaliero (Brescia, Italy). The research project was authorized by Centro di Ricerca E. Menni-Fondazione Poliambulanza.

### Isolation of Human Amniotic Mesenchymal Stromal Cells (hAMSC) and Production of Conditioned Medium (CM-hAMSC)

Human term placentas were processed immediately after birth using a previously described protocol [27]. Briefly, the amnion was manually separated from the chorion and washed extensively in PBS (Sigma, St Louis, MO, USA) containing 100U/ml penicillin and 100 mg/ml streptomycin (herein referred to as P/S, Euroclone, Whetherby, UK) and 2.5 mg/ml amphotericin B (Lonza, Basel, CH). Afterwards, the amnion was cut into small pieces (3x3 cm^2^). Amnion fragments were sterilized by a brief incubation in PBS + 2.5 % Eso Jod (Esoform, Italy) and 3 min in PBS containing 500U/ml penicillin, 500 mg/ml streptomycin, 12.5 mg/ml amphotericin B and 1.87 mg/ml Cefamezin (Pfizer, Italy). Sterilized amnion fragments were then incubated for 9 min at 37 °C in HBSS (Lonza, Basel, CH) containing 2.5U/ml dispase (Roche, Mannheim, Germany). The fragments were digested in complete RPMI 1640 medium (Cambrex, Verviers, Belgium) supplemented with 0.94 mg/ml collagenase (Roche) and 20 mg/ml DNase (Roche) for 2.5–3 hrs at 37 °C. Amnion epithelium fragments were then removed by low-g centrifugation, mobilized hAMSC were passed through a 100 μm cell strainer and collected by centrifugation. These cells are referred to as hAMSC, for human Amniotic Mesenchymal Stromal Cells, and at passage 0 (freshly isolated) are characterized by the expression of CD90 (80.5 ± 10.7 %), CD13 (84.5 ± 8.7 %), CD73 (66 ± 6 %), CD44 (57 ± 10 %), CD105 (6 ± 4 %), CD166 (9.3 ± 6.5 %), CD324 (10.4 ± 6.4 %), CD45 (7.4 ± 2.8 %), CD14 (6 ± 3 %), and negative for CD34 and CD3.

*Conditioned Medium generated from freshly isolated hAMSC*. hAMSC (obtained from amniotic membranes of at least 30 different donors) were re-suspended in an opportune volume of UltraCulture serum-free medium (Lonza, Basel, CH) supplemented with P/S, and plated in 24-well plates at 0.5×10^5^ cells/well in a final volume of 0.5 ml (referred to as CM-hAMSC). After 5-days of culture at 37 °C with 5 % CO_2_, the CM-hAMSC were collected, centrifuged at 300 g, filtered through a 0.8 μm sterile filter (Sartorius) and frozen at −80 °C until use. In order to obtain results that were less influenced by single donor variability and more representative of bioactive molecules released by hAMSC, we pooled 8 to 10 different CM-hAMSC and used them for each specific analysis.

### Purification of T-Lymphocytes and Proliferation Assays

Human peripheral blood mononuclear cells (PBMC) were obtained from heparinized peripheral blood (PB) or buffy coats (BC) of healthy donors after Ficoll–Hypaque gradient centrifugation (Sigma, St Louis, MO, USA). The purity of PBMC preparations was checked by FACS analysis to ensure low red blood cell (RBC) and polymorphonuclear (PMN) cell contaminations. T-cells were purified from PBMC by negative selection using the MACS® system (Pan T Cell Isolation Kit), (Miltenyi Biotec, Bergisch Gladbach, Germany) according to the manufacturer’s instructions. Lymphocyte proliferation was induced either by stimulating T cells (10^5^/well in 96-well-round bottom plate) by immobilized anti-CD3 (1 μg/ml OKT3) / anti-CD28 (2.5 μg/ml), or by the co-culture with irradiated allogeneic stimulator PBMC in mixed lymphocyte reactions (MLR). MLR were set up with 10^5^ effector T-lymphocytes and 10^5^ γ-irradiated (3000 cGy) allogeneic PBMC in round-bottom 96-well plates (Nunc, Roskilde, Denmark). MLR and T + anti-CD3/28 were cultured in UltraCulture medium. Responder T-cell/stimulator cell combinations were chosen on the basis of a minimum of three human leucocyte antigen (HLA) mismatches. T-cells were labeled with CFSE dye using the CellTrace™ CFSE Cell Proliferation Kit (Invitrogen, Molecular Probes, USA), according to manufacturer’s instructions. T cell proliferation was assessed by flow cytometry and is expressed as a percentage of CFSE diluting cells (Proliferative Fraction PF) or as Proliferation Index (PI). The PF represents the percent of proliferating cells and the PI is the sum of the cells in all generations divided by the number of original parent cells present at the start of the experiment. It measures the increase in cell number in culture over the course of the experiment and is calculated by using FCS express v4.07 (DeNovo Software) from a cell division model which predicts a cell doubling as a cell proliferated through each daughter generation. In order to perform FACS analysis only on the responder T-cells, γ-irradiated allogeneic stimulator PBMC were labeled using the CellVue® NIR780 Cell Labeling Kit (eBiosciences) in order to identify and exclude them from analysis. To assess the effect of CM-hAMSC on the T cell subsets, we co-cultured T cells in 50 % CM-hAMSC.

### Phenotype Analysis of T-Cell Subsets

The phenotypes of the different T cell subsets were assessed by FACS analysis by using a set of cell surface markers, intracellular transcription factors, and secreted cytokines, as reported in Table 1. After 6 days of co-culture with CM-hAMSC, the cells derived from MLR experiments were collected and centrifuged at 300 g for 5 min. To improve the efficiency of gating live cells and decrease non-specific staining of dead cells, before fixation samples were stained with Zombie NIR Live/Dead Cell Kit (eBiosciences, San Diego, USA). For fixation, 0.05 % freshly-prepared, methanol-free formaldehyde (ThermoFisher, Waltham, Massachusetts, USA) was added to the cells and incubated for 15 min at RT. Cells were then permeabilized with 0.05 % Saponin/100 mM Tris–HCl pH 7.4 for 15 min. The surface staining was carried out for 30 min at RT with the following antibodies: anti-CD4 BV421, anti-CD4 BV510, anti-CD8 BV510, anti-CD25 PerCP-Cy™5.5, anti-CD28 BV421, anti-CD45RO PE-CF594, anti-CD73 BV510, anti-CD119 PE, anti-CTLA-4 APC, anti-CD161 PE, anti-CD183 PE-Cy™7, anti-CD193 BV510, anti-CD357 APC, anti-CD294 PerCP-Cy™5.5, anti-GARP PE (all from Becton Dickinson, Franklin Lakes, New Jersey, USA) and anti-CD39 PE-Cy™7 (eBiosciences). The staining of intracellular antigens was performed by incubating the cells in 0.05 % Saponin/100 mM Tris–HCl pH 7.4 for 1 h with the following antibodies: anti-FoxP3, anti-Helios PE, anti-T-bet PE-CF594, anti-GATA3 AlexaFluor®647, anti-RORγt AlexaFluor®647, anti-TGF-β BV421 (all from Becton Dickinson, Franklin Lakes, New Jersey, USA).

**Table 1:** Markers used to identify T cell subpopulations

	Th1	Th2	Th17	Treg
Surface antigens	CD4, CD183, CD119	CD4, CD193, CD294	CD4, CD161	CD4, CD25, CD39, CD73, CD152, CD357
Transcription factors	T-bet	GATA-3	RORγt	FoxP3, Helios
Cytokines	IL-1β, IL-2, TNF-α, IFN-α, IL-12p70	IL-4, IL-5, IL-6, IL-10, IL-13	IL-17A, IL-22	TGF-β, sIL2-R

The markers described in the Table were used to identify the different T cell subsets in this study, and are divided into 3 categories: surface antigens, transcription factors, and cytokines

### Detection of Secreted Cytokines

The supernatant from MLR experiments was collected from day 1 to day 6 of culture, and the quantification of secreted cytokines was evaluated by using a multiple cytometric beads array system Human Th1/Th2/Th9/Th17/Th22 13plex FlowCytoMix kit (eBiosciences, San Diego, USA), according to the manufacturer’s instructions. The following cytokines were measured: IL-1-β, IL-2, IL-4, IL-5, IL-6, IL-9, IL-10, IL-12p70, IL-13, IL-17A, IL-22, TNF-α, and IFN-α. The levels of TGF-β and sIL2R were measured using the FlowCytoMix kit (eBiosciences, San Diego, USA), according to the manufacturer’s instructions. Samples were acquired with a FACSAria (Becton Dickinson, Franklin Lakes, New Jersey, USA) and analyzed with FlowCytomix Pro software (eBiosciences, San Diego, USA)

### Statistical Analysis

Statistical analyses were performed by means of unpaired, two-tailed *t*-tests using GraphPad Prism 6 Software (GraphPad Software, San Diego, CA, USA). Results are represented as mean ± standard deviation (SD) or standard error mean (SEM) as specified in the text. A *P*-value lower than 0.05 was considered statistically significant.

## Results

### The Effects of CM-hAMSC on T-Cell Proliferation

**Fig. 1:**
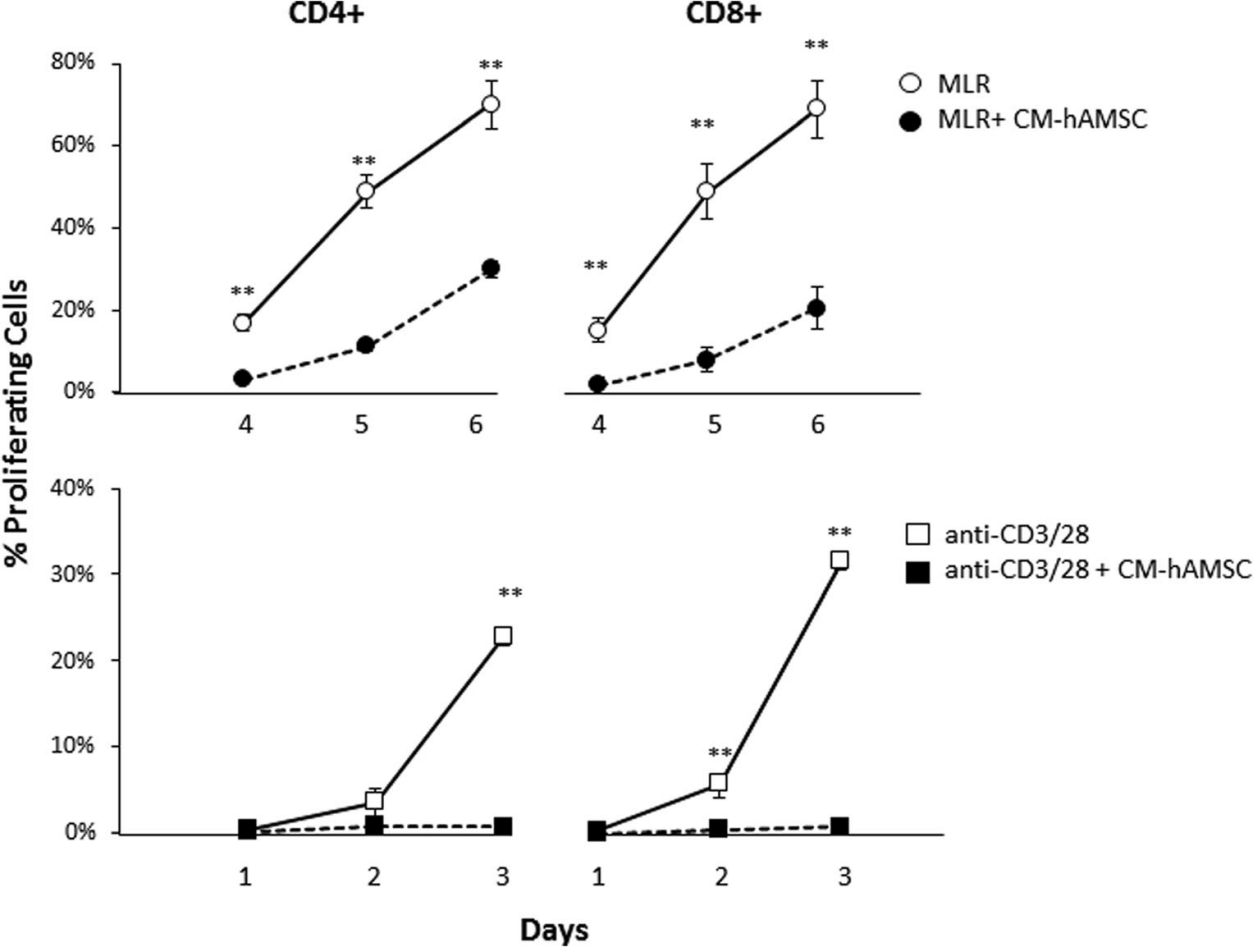
Inhibitory effects of CM-hAMSC on CD4^+^ and CD8^+^ proliferation. T cell proliferation was stimulated either with allogeneic PBMC in mixed lymphocyte reactions (MLR, circles in top panel) or by T cell receptor stimulation using anti-CD3/CD28 (squares in bottom panel). Proliferation of CD4^+^ (left panel) and CD8^+^ (right panel) was measured in the absence (empty circles/squares) or presence (black-filled circles/squares) of conditioned medium (CM-hAMSC). Data represent the mean and SD of at least four experiments. Asterisks indicate statistically significant differences between MLR-CM-hAMSC and MLR; *p < 0.05; **p < 0.01

We have previously demonstrated that conditioned medium generated from the culture of freshly isolated hAMSC (CM-hAMSC) is able to modulate lymphocyte proliferation in a dose-dependent manner [27]. Herein we assessed the paracrine effect of CM-hAMSC on the proliferation of CD4^+^ and CD8^+^ lymphocytes. As shown in Fig. 1, the CM-hAMSC suppressed the proliferation of both CD4^+^ T helper (Th) cells and CD8^+^ cytotoxic T lymphocytes (CTLs), while CM-hAMSC *per se* was not able to stimulate T cells (data not shown). The paracrine suppressive effects of CM-hAMSC were observed at different time points of the study in T cells stimulated by allogeneic PBMC (Fig. 1, top panel), and also via T cell receptor (TCR) stimulation with anti-CD3/anti-CD28 (Fig. 1, bottom panel). Specifically, at the end of co-culture with CM-hAMSC, we observed a 45 and 25 % decrease of Th proliferation in the allogeneic and TCR settings, respectively, (Fig. 1, left panel). Moreover, at the end of co-culture with CM-hAMSC we observed a 50 and 35 % decrease of CTL proliferation in the allogeneic and TCR settings, respectively, (Fig. 1, right panel).

### The Effects of CM-hAMSC on Memory/Naïve Subsets

We then set out to clarify the effects of CM-hAMSC on CD4 and CD8 subsets in terms of proliferation and phenotype. To this end, we used CFSE-labeled T cells stimulated by allogeneic PBMC (MLR) and we evaluated T cell proliferation after 6 days of culture. T cell proliferation was expressed as a percentage of CFSE diluting cells (Proliferative Fraction, PF) and by the Proliferation Index (PI).

**Fig. 2:**
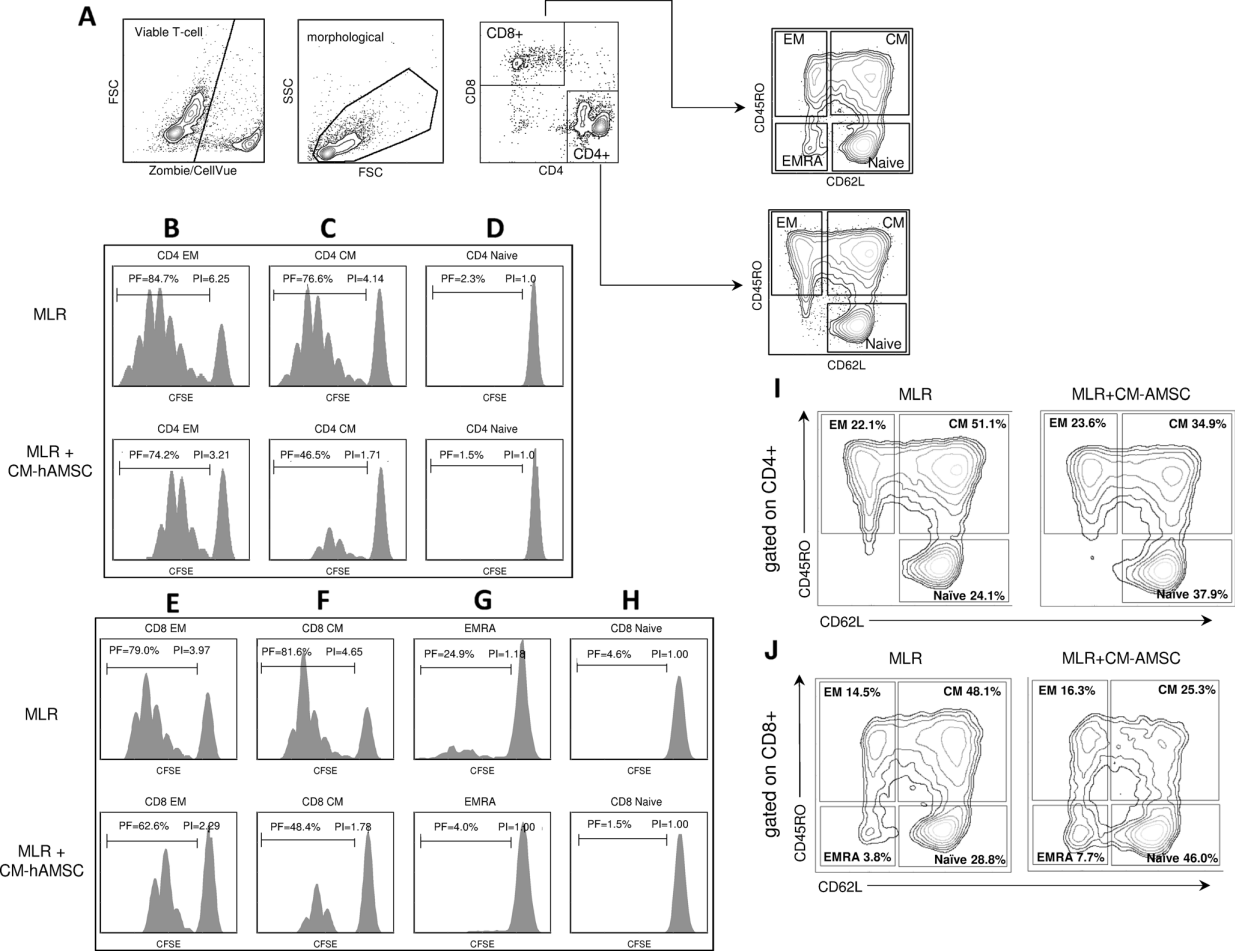
Effects of CM-hAMSC on the proliferation of CD4+ and CD8+ subsets. Panel (**A**) shows the gating strategy used for analysis. CD4 and CD8 T cell proliferation in mixed lymphocyte reactions (MLR) or co-cultured with CM-hAMSC is expressed as a percentage of CFSE diluting cells (Proliferative Fraction, PF) or by the Proliferation Index (PI). The following phenotypes were analyzed after 6 days of co-culture: (**B**) CD4 Effector Memory (EM, CD4^+^CD45RO^+^CD62L^−^); (**C**) CD4 Central Memory (CM, CD4^+^CD45RO^+^CD62L^+^); (**D**) CD4 Naïve (CD4^+^CD45RO^−^CD62L^+^); (**E**) CD8 Effector Memory (EM, CD8^+^CD45RO^+^CD62L^−^); (**F**) CD8 Central Memory (CM, CD8^+^CD45RO^+^CD62L^+^); (**G**) CD8 Effector (EMRA, CD8^+^CD45RO^−^CD62L^−^); (**H**) CD8 Naïve (CD8^+^CD45RO^−^CD62L^+^ cells). The frequency of the different subsets (reported as %) gated on the CD4 (**I**) and CD8 cells (**J**). The figure is representative of three independent experiments which showed statistically significant differences between MLR-CM-hAMSC and MLR for PF in CD4 CM, CD4 EM, CD8 CM, CD8 EM, CD8 Naïve, CD8 EMRA; and for PI in CD4 CM, CD4 EM, CD8 CM, CD8 EM

Specifically, as shown in Fig. 2, we assessed the proliferation of CD4 and CD8 T cell subsets, distinguishing their phenotype on the basis of CD45RO and CD62L as previously reported [35]: CD4 Effector Memory (CD4 EM), (CD4^+^CD45RO^+^CD62L^−^), CD4 Central Memory (CD4 CM),(CD4^+^CD45RO^+^CD62L^+^), CD4 Naïve (CD4^+^CD45RO^−^CD62L^+^), CD8 Effector Memory (CD8 EM), (CD8^+^CD45RO^+^CD62L^−^), CD8 Central Memory (CD8 CM), (CD8^+^CD45RO^+^CD62L^+^), CD8 Effector (CD8 EMRA), (CD8^+^CD45RO^−^CD62L^−^), and CD8 Naïve (CD8^+^CD45RO^−^CD62L^+^ cells). We observed that the proliferation of both CD4 Effector Memory (Fig. 2B) and CD4 Central Memory (Fig. 2C) cells are inhibited by the CM-hAMSC. Specifically, we observed a decrease in both the PF (85 to 74 % [mean ± SD: 86.75 % ± 1.45 to 79.25 % ± 4.14, *p* < 0.05]) and PI (6.25 to 3.2 [7.37 ± 0.90 to 4.14 ± 0.89, *p* < 0.01]) of the CD4 Effector Memory cells (Fig. 2B). CD4 Central Memory cells also showed a reduction in both the PF (from 77 to 47 % [79.88 % ± 2.19 to 59.33 % ± 11.37, *p* < 0.05]) and PI (4.14 to 1.71 [4.54 ± 0.45 to 2.54 ± 0.89, *p* < 0.01]), (Fig. 2C). On the other hand, the CD4^+^ Naïve cells did not proliferate and showed a resting behavior both prior to (PF: 2.3 %, PI: 1.0 [PF: 1.75 % ± 0.37, PI: 1.0 ± 0.0]) and after (PF: 1.5 %, PI: 1.0 [PF: 1.53 % ± 0.24, PI: 1.0 ± 0.0]) culture with CM-hAMSC (Fig. 2D). Similar effects were seen on CD8 subsets where co-culture with CM-hAMSC inhibited the proliferation of CD8 Effector Memory cells (PF: 79 % vs. 63 %, PI: 4 % vs. 2.3 % [PF: 80.41 % ± 1.16 vs. 69.91 % ± 4.08, *p* < 0.01; PI: 5.01 ± 0.93 vs. 2.71 ± 0.58, *p* < 0.01], Fig. 2E), CD8 Central Memory cells (PF: 82 % vs. 48 %, PI 4.65 % vs. 1.8 %, [PF: 86.89 % ± 3.25 vs. 62.91 % ± 9.37, *p* < 0.01; PI: 7.55 ± 2.19 vs. 2.46 ± 0.66, *p* < 0.01], Fig. 2 F), and CD8 Effector (PF: 25 % vs. 4 %, PI 1.2 % vs. 1 %, [PF: 16.57 % ± 7.62 vs. 5.45 % ± 1.45, *p* < 0.05; PI: 1.08 ± 0.11 vs. 1.00 ± 0.00, *p* < 0.01], Fig. 2G). As observed in the CD4^+^ cells, CM-hAMSC had no effect on the proliferation of CD8 Naïve cells, which had a resting behavior prior to and after co-culture (PF: 4.6 % vs. 1.5 %, PI 1 % vs. 1 %, [PF: 3.21 % ± 1.15 vs. 1.31 % ± 0.60, *p* < 0.05; PI: 1.00 ± 0.00 vs. 1.00 ± 0.00], Fig. 2H).

On the basis of the observed inhibitory ability, we also evaluated how the CM-hAMSC influenced the relative content/frequency of each T-cell subset described above. Regarding the CD4 positive cells, we observed a reduction in the Central Memory compartment, no variation in the Effector Memory compartment, and a relative increase of Naïve population (Fig. 2I). Similarly, within the CD8^+^ population, we observed a decrease of the Central Memory and a relative increase of the Naïve compartment (Fig. 2J). Finally, we observed that in the presence of CM-hAMSC, the percentage of CD8 Effector Memory did not change while that of CD8 Effector increased in the presence of CM-hAMSC (Fig. 2J).

**Fig. 3:**
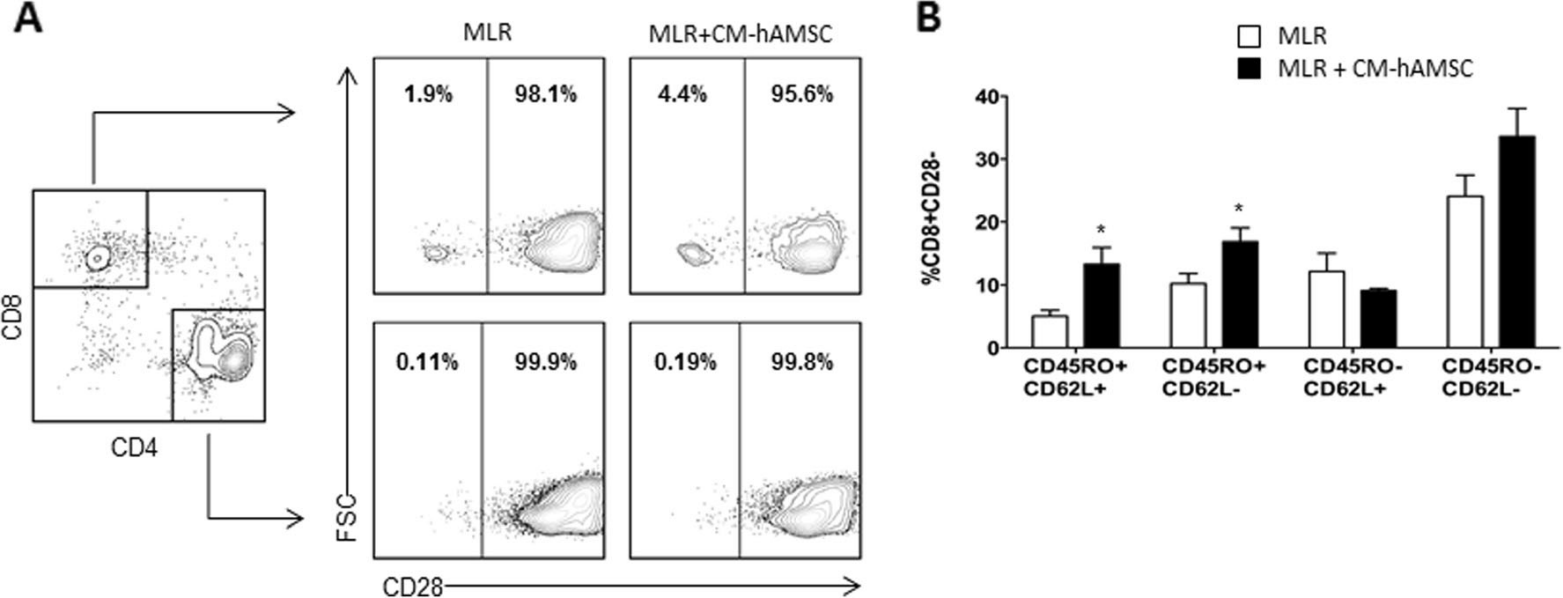
CM-hAMSC increases the CD28- population within CD8+ subsets. Flow cytometry analysis was performed on allostimulated T cells in presence or absence of CM-hAMSC after 6 days of co-culture. (**A**) The percentage of cells expressing CD28 was evaluated in the total CD8^+^ cells (upper panels) or in the CD4^+^ cells (bottom panels). (**B**) The CD45RO and CD62L positivity was evaluated by gating on CD8^+^CD28^−^ cells. Data represent the mean and SD of at least 3 experiments. Asterisks indicate statistically significant differences between MLR-CM-hAMSC and MLR; *p < 0.05

We performed further analysis of T-cell populations based on their CD28 co-stimulatory molecule expression. It is known that CD8^+^ cells can be distinguished into cytotoxic CD8^+^CD28^+^ and suppressor/regulatory CD8^+^CD28^−^ subsets [36]. As shown in Fig. 3A (upper panel), the presence of CM-hAMSC increased the frequency of CD8^+^CD28^−^ T cells, while no difference was observed after culture with CM-hAMSC in the CD4^+^CD28^−^ population. To further characterize the phenotype of the CD8^+^CD28^−^ cells, we also evaluated the expression for CD45RO and CD62L markers. Even though most of CD8^+^CD28^−^ cells showed a Naïve phenotype (CD45RO^−^CD62L^+^), (Fig. 3B), we observed a significant increase in the percentage of the CD8^+^CD28^−^ population induced by CM-hAMSC within both the Effector Memory (CD45RO^+^CD62L^−^) and the Central Memory (CD45RO^+^CD62L^+^) compartments (Fig. 3B).

### The Effects of CM-hAMSC on T Helper (Th) Differentiation

**Fig. 4:**
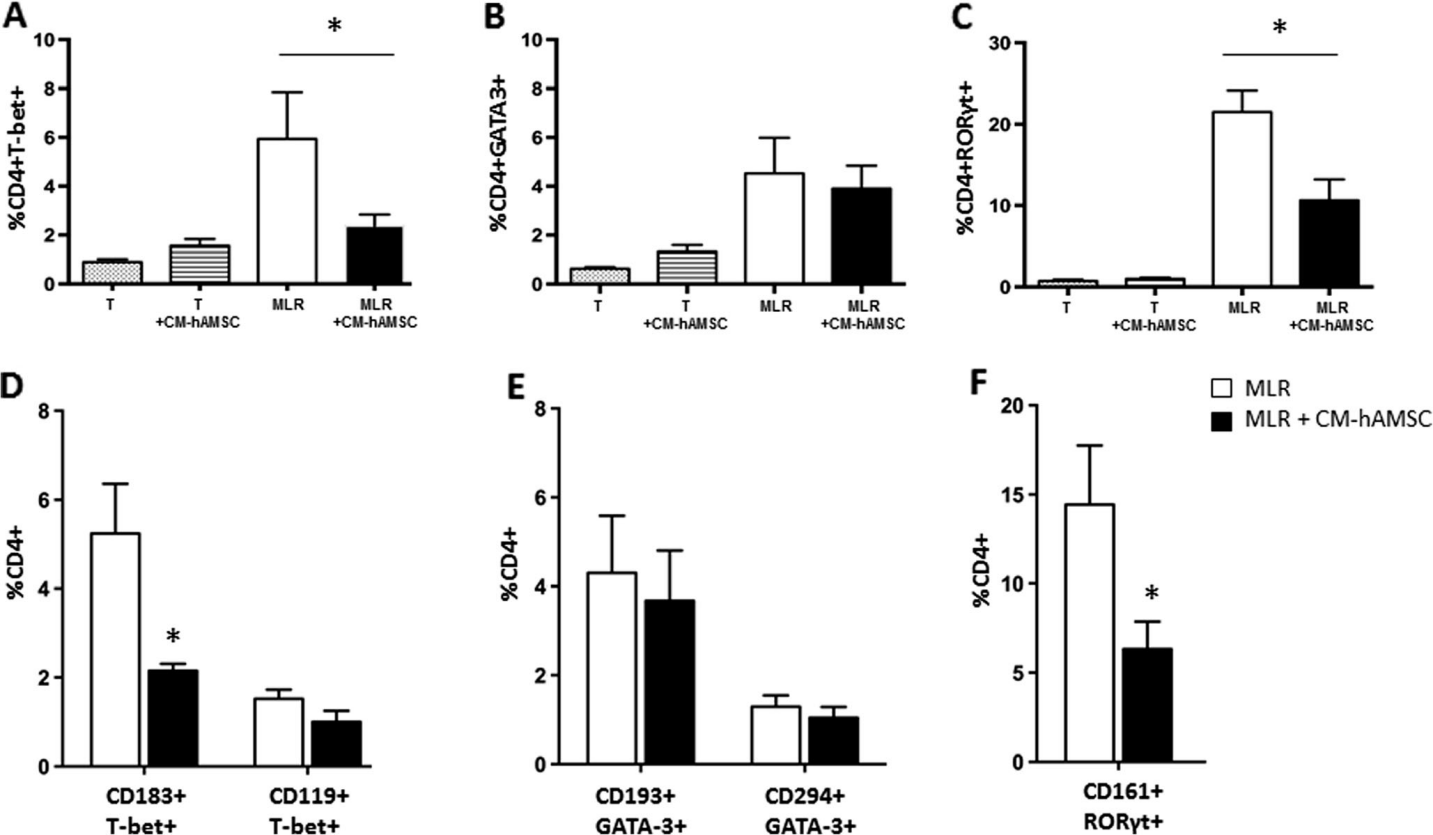
Effects of CM-hAMSC on the T helper polarization. The expression of the following T helper transcription factors: T-bet^+^ (Th1) (**A**) GATA-3^+^ (Th2) (**B**) RORγt^+^ (Th17) (**C**) was evaluated on unstimulated T-cells in absence (T, gray-dotted bars) or presence (T + CM-hAMSC, lined bars) of CM-hAMSC, and on alloreactive CD4+ cells in the absence (white bars) or presence (black bars) of CM-hAMSC. The phenotypes of the different CD4 T helper subsets were assessed by double positive populations for both Th-specific transcription factors and surface markers: T-bet^+^CD183^+^ and T-bet^+^CD119^+^ (Th1) (**D**), GATA-3^+^CD193^+^ and GATA-3^+^CD294^+^ (Th2) (**E**), RORγt^+^CD161^+^ (Th17) (**F**), as reported in Table 1. Data represent the mean and SD of at least five experiments. Asterisks indicate statistically significant differences between MLR-CM-hAMSC and MLR; *p < 0.05

To assess the effects of bioactive molecules derived from hAMSCs on the polarization of Th cells, we evaluated the phenotype of CD4^+^ cells after 6 days of co-culture. As shown in Fig. 4, CM-hAMSC by itself did not induce significant changes in the percentage of cells positive for Th transcription factors (T-bet, GATA-3, RORγt) in unstimulated T cells. In the MLR setting, CM-hAMSC significantly reduced CD4^+^T-bet^+^ and CD4^+^RORγt^+^ cells (Fig. A and 4C) whilst having no effect on CD4^+^GATA3^+^ cells (Fig. 4B). Moreover, CM-hAMSC caused a 60 % reduction in T-bet^+^CD183^+^ cells (Fig. 4D, *p* < 0.05), which are markers associated to Th1 cells. Conversely, co-culture with CM-hAMSC had no effect on the T-bet^+^CD119^+^ subpopulation, also associated to Th1 cells (Fig. 4D). No changes were observed also for the Th2 population, represented by GATA3^+^CD193^+^ and GATA3^+^CD294^+^ (Fig. 4E). Finally, co-culture with CM-hAMSC caused a 56 % reduction in RORγt^+^CD161^+^, a marker associated to Th17 cells (Fig. 4 F, *p* < 0.05). Taken together, these data demonstrate that the CM-hAMSC-mediated inhibition of alloreactive T lymphocytes is associated also with modulation of the Th1 and Th17 pathway.

### The Effects of CM-hAMSC on T Regulatory (Treg) Cells

**Fig. 5:**
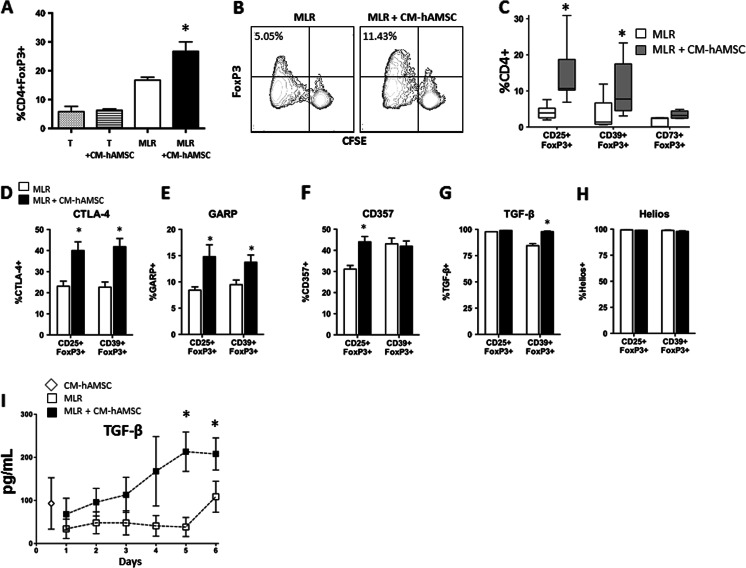
CM-hAMSC induces T lymphocytes with regulatory phenotype. (**A**) The percentage of CD4^+^FoxP3^+^ cells in unstimulated T cells in absence (T, gray-dotted bars) or presence (T + CM-hAMSC, lined bars) of CM-hAMSC, and in stimulated (MLR) T cells in absence (white bars) or presence (black bars) of CM-hAMSC. Data represent mean and SD of 5 individual experiments. (**B**) The percentage of proliferating and FoxP3-positive cells in the presence or absence of CM-hAMSC. The plot is representative of 3 experiments. (**C**) CD4 Treg phenotypes were assessed by double positive populations for both FoxP3 and surface markers (CD25, CD39, CD73). Box and whispers plots were generated using the Tukey method. CD25 Treg and CD39 Treg were characterized by additional surface markers CTLA-4 (**D**), GARP (**E**), CD357 (**F**), and intracellular markers such as TGF-β (**G**) and Helios (**H**). The release of TGF-β during 6 days co-culture was evaluated as described in Materials and Methods, the graph represents the mean and standard error mean (SEM) of at least four experiments (**F**). Asterisks indicate statistically significant differences between MLR + CM-hAMSC and MLR; *p < 0.05

Based on the evidence of Th polarization exerted by CM-hAMSC, we sought to analyze the phenotype of induced T regulatory (Treg) cells. To this aim, we assessed alloreactive CFSE–labeled responder T cells after 6 days of co-culture in presence of CM-hAMSC by evaluating the percentage of CFSE-diluting (i.e. dividing) T cells. Interestingly, we observed that CM-hAMSC intensively modulates the Treg compartment (Fig. 5). First, we observed that CM-hAMSC by itself was not able to induce the expression of FoxP3, but in the MLR setting CM-hAMSC significantly increased CD4^+^FoxP3^+^ cells (Fig. 5A). We also observed that the induction of FoxP3 cells was restricted to proliferating cells (Fig. 5B). As shown in Fig. 5C, the CM-hAMSC intensively modulates the Treg compartment by inducing a five-fold increase of CD25^+^FoxP3^+^ cells. We also evaluated the expression of CD39 and CD73 in Treg and found that the percentage of CD39-expressing Treg in the CD4^+^ population markedly increased in the presence of CM-hAMSC (Fig. 5C). We observe an increase also of the CD73^+^ population even though it did not reach significance (Fig. 5C). Further characterization of cell surface antigens expressed by the CM-hAMSC-induced Treg cells demonstrated different patterns of expression when compared with Treg present in the MLR itself. In particular, we observed an increase of CTLA-4^+^ (Fig. 5D) and GARP^+^ (Fig. 5E) cells within both CD4^+^CD25^+^FoxP3^+^ and CD4^+^CD39^+^FoxP3^+^ (Fig. 5E). TGF-β was highly expressed by both CD25^+^ and CD39^+^ FoxP3^+^ Treg cells, but the percentage of TGF-β cells increased significantly by CM-hAMSC only in the CD39^+^ FoxP3^+^ Treg (Fig. 5G). Conversely, we observed a significant increase of CD357^+^ (AITR/GITR) cells only in the CD4^+^CD25^+^FoxP3^+^ population in presence of CM-hAMSC (Fig. 5F). Finally, both CD4^+^CD25^+^FoxP3^+^ and CD4^+^CD39^+^FoxP3^+^ cells were all positive for Helios (Fig. 5H). To confirm the induction of Treg cells, we analyzed the release of TGF-β during 6 days of co-culture of alloreactive T cells in presence of CM-hAMSC. As shown in Fig. 5I, the CM-hAMSC induced the secretion of TGF-β starting from 3 days of co-culture, and this induction was significant and consistent over time (*p* < 0.05).

### CM-hAMSC Modifies the Secretion of Th-Cytokines

**Fig. 6:**
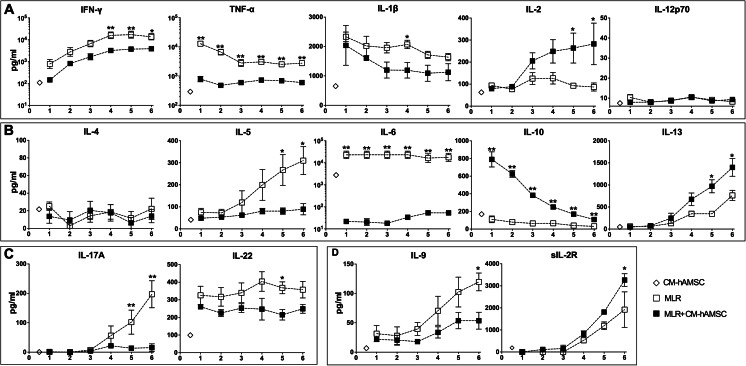
CM-hAMSC modulates the secretion of Th cytokines. The secretion of cytokines specific for the different T-cell subsets was evaluated each day during 6 days of co-culture in absence (white squares) or presence (black squares) of CM-hAMSC. The amount of cytokine produced by the CM-hAMSC itself was measured on day one only and is represented by a white diamond. The quantification of (**A**) Th1 cytokines (IFN-γ, TNFα, IL-1β, IL-2, IL-12p70), (**B**) Th2 cytokines (IL-4, IL-5, IL-6, IL-10, IL-13), (**C**) Th17 cytokines (IL-17A, IL-22), (**D**) IL-9 and sIL-2R were evaluated as described in Materials and Methods. Data represent the mean and standard error mean (SEM) of at least three experiments. Asterisks indicate statistically significant differences between MLR-CM-hAMSC and MLR; *p < 0.05; **p < 0.01

After having studied which Th cells were directly inhibited by CM-hAMSC, in order to give further insight into the function of the different T subsets, we studied their cytokine profile. To this aim, we analyzed cytokine release for 6 days during co-culture with alloreactive T cells. To assess the modulation of secreted molecules in the culture medium, we compared the level of cytokines released from alloreactive T cells in presence or absence of CM-hAMSC. We also considered the initial amount of cytokine produced by the CM-hAMSC itself (white diamond in Fig. 6). As shown in Fig. 6, we analyzed a panel of cytokines specific for the different T-cell subsets: Th1 (IFN-γ, TNFα, IL-1β, IL-2, IL-12p70), Th2 (IL-4, IL-5, IL-6, IL-10, IL-13), Th17 (IL-17A, IL-22), and Th9 (IL-9) (Table 1). For all cytokines analyzed except IL-6, levels in the CM-hAMSC per se were below levels detected in MLR on day 1 (Fig. 6). In the presence of CM-hAMSC, the secretion of the pro-inflammatory Th1-cytokines TNFα and IFNγ was strongly inhibited starting from 24 to 48 h respectively, and this inhibition remained constant over time (Fig. 6A). To a lesser extent, IL-1β also decreased starting from 48 h up to 5 days after the addition of CM-hAMSC (Fig. 6A). Interestingly, the IL-2 secretion increased over time in the presence of CM-hAMSC, while the levels of IL-12p70 were not affected by the addition of CM-hAMSC, which remained low throughout the time course (Fig. 6A). In regards to the Th2-cytokines, the secretion of IL-5 and IL-6, was markedly reduced starting from 24 h of co-culture with CM-hAMSC (Fig. 6B). As we have previously observed [27], the CM-hAMSC per se contained high levels of IL-6 (Fig. 6B). Conversely, the presence of CM-hAMSC induced the secretion of IL-10 starting from 24 h of co-culture, and this induction decreased over time, while the CM-hAMSC had no effect on IL-4 secretion over time (Fig. 6B). The CM-hAMSC induced an increase in the secretion of IL-13 starting from 72 h after co-culture/activation (Fig. 6B). The release of Th17-cytokines (IL-17A and IL-22) and IL-9, a Th9-related cytokine, were inhibited by the CM-hAMSC, and this inhibition remained consistent throughout the 6-day observation period (Fig. 6C and D). Finally, sIL-2R significantly increased in the presence of CM-hAMSC starting from 3 days after co-culture and continuing up to the end (day 6), (Fig. 6D, *p* < 0.05).

## Discussion

Increasing evidence indicate that derivatives from the human amniotic membrane, such as patches, cells, and conditioned medium derived from these cells, exert therapeutical effects in diseases associated with altered inflammatory processes [8] or in autoimmune disorders [6]. The lack or very low engraftment of transplanted cells, and the evidence that conditioned medium *per se* is effective, supports the notion that the therapeutic effect is due to bioactive molecules released from hAMSC that act through a paracrine/endocrine mechanism.

A likely explanation of the beneficial effects exerted by hAMSC is associated to the immunomodulatory potential of these cells, a characteristic identified previously in MSC from other sources, such as bone marrow [37–40]. Indeed, it has been extensively reported that hAMSC can inhibit T cell proliferation induced by alloantigens, T-cell receptor cross-linking, or mitogens in vitro [13–15, 17, 19] and can inhibit the generation, maturation and function of monocyte-derived dendritic cells (DCs) [18, 41]. The confirmation that the molecules released from cells are the key players in their immunomodulatory effect comes from the observation that the conditioned medium obtained from the culture of both AM patches and hAMSC inhibit T cell proliferation [27], inhibit the differentiation of monocytes towards DCs and induce a shift toward M2-like macrophages [28, 29]. T cells have a prominent role in immune regulation, and polarization of the different T-cell subsets plays an important role in controlling the mechanisms of immune response in phenomena like acute and chronic inflammation and autoimmune responses. Since until now reports which provide evidence that hAMSC act on T cells are mainly based on the effects on total T cells and often limited to the proliferative parameters, we set out to perform a detailed study on the effects of conditioned medium from hAMSC on both CD4 and CD8 subsets and on different Th subsets. To this aim, we analyzed cell proliferation, alterations in phenotype, and cytokine production in a time-course response.

First, we observed that conditioned medium derived from hAMSC, when cultured without inflammatory stimuli, suppresses the proliferation of both CD4^+^ Th cells and CD8^+^ cytotoxic T lymphocytes (CTLs) stimulated by allogeneic PBMC, and also via T cell receptor (TCR) stimulation with anti-CD3/anti-CD28. This supports our previous observations that CM derived from amnion possess anti-proliferative effect in absence of stimulating culture conditions [27]. This is in contrast to BM-MSC which possess an anti-proliferative ability only when cultured in the presence of activating stimuli, such as IL-1β, TNF-α or IFN-γ [30, 42, 43].

The differentiation of T cells into effector and memory subsets is one of the key aspects of T cell mediated immunity. We therefore characterized the effect of hAMSC on the T-cell response of Naïve and Memory T cells and demonstrate that CM-hAMSC inhibited the proliferation of both CD4/CD8 Effector Memory and CD4/CD8 Central Memory cells, while no variation was observed regarding the proliferation of the CD4/CD8 Naïve T-cell population. Others have shown that bone marrow MSC are able to equally inhibit the proliferation of Memory and Naïve T cells using HY peptide-stimulated splenocytes from transgenic HY-TCRhigh mice [44]. The relative increase we observed in the percentage of Naïve cells (CD45RO^−^CD62L^+^) after CM-hAMSC co-culture can be justified by the decrease observed in the other subsets.

Within the CD8^+^ population (which drastically decreased in the presence of CM-hAMSC), we observed an increase of the CD8^+^CD28^−^ population. This can be explained by the preferential survival of this population, as manifested by the fact that we observed a relative increase of CD8^+^CD28^−^ within the Effector Memory, Central Memory, and EMRA compartments. Interestingly, CD8^+^CD28^−^ T cells have been reported as T regulatory cells [45, 46]. Indeed, CD8^+^CD28^−^ T cells have been shown to be accountable for regulatory functions associated to disease amelioration in an autoimmune mouse model [47]. Moreover, they have been reported to be able to down-regulate the Th reactivity by suppression of antigen-presenting cells [48], and to be responsible for the inhibition of both T-cell proliferation and CTL function [49]. The effect of MSC on the CD8^+^CD28^−^ population is still a matter of debate. Indeed, it has been shown that MSC from adipose tissue induce an inhibition of CD8^+^CD28^−^ cells [50], while others, in accordance with our data, have shown that MSC from bone marrow induce an increase of this population thus contributing to the attenuation of refractory dry eye secondary to chronic graft-versus-host-disease [51].

Within the CD4 population we were able to confirm the evident anti-inflammatory properties of hAMSC. Indeed our results showed that CM-hAMSC induced the inhibition of Th1 (Tbet^+^CD183^+^) and Th17 (RORγt^+^CD161^+^) subset proliferation, and down-regulated pro-inflammatory Th1 cytokines IFN-γ, TNFα, and IL-1β, and Th17 cytokines such as IL-17A and IL-22. Even though CM-hAMSC did not influence Th2 (GATA3^+^/CD193^+^ or GATA3^+^/CD294^+^) cell expansion, the release of Th2 cytokines, such as IL-5 and IL-6, was significantly reduced in the presence of CM-hAMSC.

Treg, a subpopulation of CD4^+^ T cells commonly identified by the expression of Forkhead box P3 (FoxP3) transcription factor, are key players in the mechanisms that are evoked to control the immune response. The two main subsets of Treg are natural Treg, which are thymus-derived and specific for self-antigens, and adaptive/induced Treg which can be generated from Naïve CD4^+^ T cells in peripheral lymphoid tissues following inflammatory stimuli [52, 53]. Both bone marrow [37, 54, 55] and adipose tissue [56, 57]-derived MSC have been extensively studied for their capacity to induce Treg induction.

The major cytokines responsible for inducing the differentiation of iTregs are IL-2 and TGF-β [58]. In presence of CM-hAMSC, we observed an increase of IL-2 and soluble form of CD25 (sCD25 or sIL-2R), and also of TGF-β. These observations further support T cell differentiation toward the Treg phenotype in the presence of CM-hAMSC. Tregs are able to secrete anti-inflammatory cytokines such as TGF-β, IL-10, IL-13 [59, 60] and these cytokines are known to be critical factors involved in the suppression of the pro-inflammatory cytokine response. Indeed, we observed an increase of the production of TGF-β and IL-13 during the co-culture of allogeneic activated T cells with CM-hAMSC. In addition to CD4^+^CD25^+^FoxP3^+^ Tregs, we also observed an increase in Tregs expressing CD39, suggesting that the adenosynergic pathway, which has functional relevance for cellular immunoregulation [61–63], could also be involved in the immunomodulatory functions exerted by CM-hAMSC.

Furthermore, in co-cultures with CM-hAMSC, CD4^+^CD25^+^FoxP3^+^ and CD4^+^CD39^+^FoxP3^+^ Tregs showed an increase in percentage of cells positive for Cytolytic T lymphocyte-associated antigen (CTLA)-4 and Glycoprotein A Repetitions Predominant (GARP), which have been shown to selectively identify activated human FoxP3+ regulatory T cells [64]. CTLA-4 has been shown to participate in Treg-mediated suppression by inhibition of dendritic cell (DC)-mediated T-cell stimulation [65, 66]. GITR appears to control DC and monocyte development and in its absence, mice develop aggravated chronic enterocolitis via an imbalance of colitogenic Th1 cells and Treg cells [67]. Taken together, these data strongly demonstrate that CM-hAMSC not only induces upregulation of the Treg population, but also induces Treg functions as shown by the altered activation of specific surface molecules that could contribute to the control of the immune suppression.

Interestingly, we demonstrated up-regulation of Treg in the culture of allogeneic activated T cells in the presence of conditioned medium derived from unstimulated hAMSC culture. This is in line with what we previously observed for CM on total T cell populations [27], and our current observation regarding CD4 and CD8 subpopulations, as well as for Th1 or Th17 subsets. These findings also support the in vivo data showing upregulation of Treg in autoimmune disorders, such as that seen with PBMC from patients with rheumatoid arthritis after addition of either hAMSC or CM-hAMSC [6]. This is in contrast to MSC derived from bone marrow, whereby stimulation with inflammatory cytokines, such as TNFα or IFNγ, are required in order to have immune regulatory effects and specifically induce Treg [68, 69].

In conclusion, this study provides new insights regarding the immune-modulating mechanism of hAMSC associated to the therapeutical effect observed in pre-clinical in vivo models and hypothesized to constitute the basis for their clinical application. Altogether, these results reinforce the potential use of these cells, and in particular their conditioned medium, which could constitute a cell-free treatment in diseases correlated to an altered inflammatory response.

## Abbreviations

MSC: Mesenchymal stromal cells
hAM: Human amniotic membrane
hAMSC: Human amniotic mesenchymal stromal cells
BM-MSC: Bone marrow mesenchymal stromal cells
CM-hAMSC: Conditioned Medium derived from the culture of hAMSC
PF: Proliferative Fraction
PI: Proliferation Index
CM: Central Memory
EM: Effector Memory
EMRA: Effector Memory RA
Treg: T regulatory cells
GATA3: GATA-binding protein 3
GITR: glucocorticoid-induced TNFR-related protein
RORγt: Retinoic acid-related orphan receptor gamma t
FoxP3: Forkhead box P3
GARP: Glycoprotein A Repetitions Predominant
T-bet: T-box transcription factor TBX21
